# Why is malaria associated with poverty? Findings from a cohort study in rural Uganda

**DOI:** 10.1186/s40249-016-0164-3

**Published:** 2016-08-04

**Authors:** Lucy S. Tusting, John Rek, Emmanuel Arinaitwe, Sarah G. Staedke, Moses R. Kamya, Jorge Cano, Christian Bottomley, Deborah Johnston, Grant Dorsey, Steve W. Lindsay, Jo Lines

**Affiliations:** 1 Department of Disease Control, London School of Hygiene & Tropical Medicine, London, UK; 2 Big Data Institute, Nuffield Department of Medicine, University of Oxford, Oxford, UK; 3 Infectious Diseases Research Collaboration, Kampala, Uganda; 4 Department of Clinical Research, London School of Hygiene and Tropical Medicine, London, UK; 5 School of Medicine, Makerere University College of Health Sciences, Kampala, Uganda; 6 MRC Tropical Epidemiology Group, London School of Hygiene and Tropical Medicine, London, UK; 7 Department of Economics, SOAS, University of London, London, UK; 8 Department of Medicine, University of California, San Francisco, USA; 9 School of Biological and Biomedical Sciences, Durham University, Durham, UK

**Keywords:** Malaria, Socioeconomic, Poverty, Development, Housing, Wealth index, Uganda

## Abstract

**Background:**

Malaria control and sustainable development are linked, but implementation of ‘multisectoral’ intervention is restricted by a limited understanding of the causal pathways between poverty and malaria. We investigated the relationships between socioeconomic position (SEP), potential determinants of SEP, and malaria in Nagongera, rural Uganda.

**Methods:**

Socioeconomic information was collected for 318 children aged six months to 10 years living in 100 households, who were followed for up to 36 months. Mosquito density was recorded using monthly light trap collections. Parasite prevalence was measured routinely every three months and malaria incidence determined by passive case detection. First, we evaluated the association between success in smallholder agriculture (the primary livelihood source) and SEP. Second, we explored socioeconomic risk factors for human biting rate (HBR), parasite prevalence and incidence of clinical malaria, and spatial clustering of socioeconomic variables. Third, we investigated the role of selected factors in mediating the association between SEP and malaria.

**Results:**

Relative agricultural success was associated with higher SEP. In turn, high SEP was associated with lower HBR (highest versus lowest wealth index tertile: Incidence Rate Ratio 0.71, 95 % confidence intervals (*CI*) 0.54–0.93, *P* = 0.01) and lower odds of malaria infection in children (highest versus lowest wealth index tertile: adjusted Odds Ratio 0.52, 95 % *CI* 0.35–0.78, *P* = 0.001), but SEP was not associated with clinical malaria incidence. Mediation analysis suggested that part of the total effect of SEP on malaria infection risk was explained by house type (24.9 %, 95 % *CI* 15.8–58.6 %) and food security (18.6 %, 95 % *CI* 11.6–48.3 %); however, the assumptions of the mediation analysis may not have been fully met.

**Conclusion:**

Housing improvements and agricultural development interventions to reduce poverty merit further investigation as multisectoral interventions against malaria. Further interdisplinary research is needed to understand fully the complex pathways between poverty and malaria and to develop strategies for sustainable malaria control.

**Electronic supplementary material:**

The online version of this article (doi:10.1186/s40249-016-0164-3) contains supplementary material, which is available to authorized users.

## Multilingual abstracts

Please see Additional file 1 for translations of the abstract into the six official working languages of the United Nations.

## Background

As attention shifts to the Sustainable Development Goals, malaria control is at a pivotal juncture. The past 15 years have seen a 37 % fall in annual global incidence [1], largely driven by the scale-up of long-lasting insecticide-treated nets (LLINs), indoor residual spraying and improved case management [2]. While these are highly effective interventions, malaria is closely associated with poverty and underdevelopment. Therefore, in the long-term, there is arguably a need for more sustainable control strategies that embrace non-health sectors, including agriculture, water and sanitation, and housing [3]. Historically, social and environmental changes contributed to malaria elimination in the USA and Europe [4]. Reflecting this, the 2013 *Multisectoral Action Framework for Malaria* outlined practical steps to target the social and environmental determinants of malaria [5]. More recently the World Health Organization’s 2015 *Global Technical Strategy for Malaria* and the complementary Roll Back Malaria action plan both seek to link malaria control with sustainable development [6].

Yet despite the potential value of a multisectoral approach to malaria, our understanding of how to target such intervention remains poor [5]. Research on socioeconomic risk factors for malaria has proliferated in the past decade and studies in a range of African settings have observed that the odds of malaria infection are on average doubled in children with the lowest socioeconomic position (SEP) (as measured by household wealth index scores or parents’ educational status or occupation), compared with children with the highest SEP within the same community [3]. However, to our knowledge, no published studies have explicitly explored the underlying causal pathways between household-level poverty and malaria. While there is evidence of reverse causality from malaria to poverty [7, 8], wealth in turn can help to protect against malaria. This protection may stem from better access to health care, LLIN coverage, treatment-seeking behaviour, housing quality and food security among other variables [9–11], yet the relative contribution of these factors remains unknown. Furthermore, few malaria studies have considered the determinants of rural poverty itself, limiting the evidence on the potential overlap between development initiatives and malaria control [5]. Here we aim to narrow these knowledge gaps through a novel, interdisciplinary investigation of the association between SEP, its determinants, and malaria among children in Nagongera, Uganda, a rural area with high malaria transmission. To our knowledge, the present study is the first explicitly to investigate factors mediating the relationship between SEP and malaria.

## Methods

### Study site

The study was carried out between August 2011 and September 2014 in Nagongera sub-country, Tororo, Uganda (00°46’10.6”N, 34°01’34.1”E). Malaria transmission is intense with two annual peaks following the two rainy seasons (March to May and August to October). During 2011–2013 the estimated annual *Plasmodium falciparum* entomological inoculation rate was 125 [12] and malaria incidence in children was 2.8 episodes per person year at risk [13]. 36 % of households have at least one LLIN per two residents but IRS is not currently done [13]. Smallholder agriculture is the primary livelihood source. Average gross national income per capita in Uganda in 2014 was US$ 670 (current prices) [14].

### Cohort study

This study was part of a cohort study, described elsewhere, which was designed to compare temporal changes in malaria incidence from the cohort with temporal changes in malaria test positivity rate from health facility based surveillance [12, 13]. All children aged six months to 10 years and their primary caregivers (individuals with primary responsibility for each child’s care) were enrolled in August-September 2011 from 100 households randomly selected from an enumeration census of all households in the sub-county. Recruitment was dynamic such that eligible children reaching six months were enrolled and children reaching 11 years were withdrawn. Households with no remaining study participants were withdrawn and replaced. Participants were followed for all healthcare needs at the study clinic for seven days a week over 36 months, until September 2014. All study participants were provided a LLIN at enrollment and compliance was >99 % by self-report at the time of routine clinic visits.

New episodes of malaria were diagnosed by passive case detection. Individuals presenting with a fever or history of fever within the past 24 h with a positive blood smear were treated with artemether-lumefantrine (uncomplicated malaria) or quinine (complicated malaria). In addition, participants were invited to make a routine visit to the study clinic every three months. At each of these visits, a thick blood smear was taken to assess for parasitaemia. Thick and thin blood smears were stained with 2 % Giemsa and read blind. Blood smears were considered negative when the examination of 100 high power fields did not reveal asexual parasites. All blood slides were read twice and discrepancies resolved by a third reviewer. In addition, all positive blood smears with a parasite densities ≤20 000/μl based on the field readings were re-read by an expert microscopist based in Kampala and had to be confirmed to be considered positive in the final analyses.

Indoor human biting rate (HBR), the number of adult female *Anopheles* caught per house per night, was measured by one monthly catch per home using a Centers for Disease Control and Prevention (CDC) light trap (Model 512; John W. Hock Company, Gainesville, FL). CDC light traps were positioned 1 m above the floor at the foot of the bed, where a study participant slept under a LLIN, and were set from 7.00 pm until 7.00 am.

### Conceptual framework

**Fig. 1:**
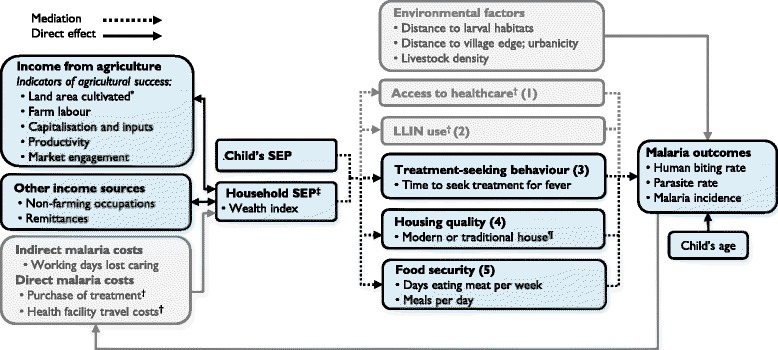
Conceptual framework for the relationship between relative agricultural success, socioeconomic position (SEP) and malaria in Nagongera, Uganda. In sub-Saharan Africa, the odds of malaria infection are on average halved in children with the highest socioeconomic position (SEP) within a community, compared to children with the lowest SEP [3]. Household SEP may be approximated using a wealth index.^‡^ Wealthier children are hypothesised to have a lower risk of malaria due, among other factors, to: (1) greater disposable income, that makes prophylaxis, treatment and transport to clinics more affordable and therefore improves access to health care [9], (2) greater ownership and use of LLINs [9], (3) improved treatment-seeking behaviour among caregivers [9], (4) better housing, which lowers the risk of exposure to malaria vectors indoors [11, 16] and (5) greater food security, which reduces undernutrition and protein-energy malnutrition and possibly susceptibility to malaria infection and progression to severe disease [10] (though the evidence is inconsistent [20]). Modern houses^¶^ were defined as those with cement, wood or metal walls; a tiled or metal roof and closed eaves. All other houses were classified as traditional. Access to healthcare^†^ and LLIN use^†^ were not hypothesised to be associated with SEP in this study population, since LLINs and all healthcare were provided by the study free of charge, but wealthier households were hypothesised to seek treatment more promptly than poorer households. Other household-level risk factors for malaria include distance to larval habitats, distance to village periphery, urbanicity and the density of livestock nearby, which were outside the scope of this study. In turn, malaria imposes costs that can cause poverty [7, 8], but this feedback loop was not analysed in this study. Heterogeneity in SEP is hypothesised to be driven largely by relative success in smallholder agriculture, since agriculture is the primary livelihood source in Nagongera (Box 1). There are many other determinants of SEP that are well studied outside the health sphere [18, 24], but we include here only non-agricultural income and access to remittances. Land area cultivated^*^ is included as an indicator of relative agricultural success, but may also be a determinant of relative agricultural success among other factors which are outside the scope of this study. This conceptual framework is not an exhaustive representation of all malaria risk factors, confounders, mediators and causal associations, but includes only those analysed in this study. The conceptual framework adds greater complexity to those by de Castro [8] and Somi [7], which primarily demonstrate bi-directionality, while the present study is chiefly interested in dissecting the strands of the poverty-to-malaria direction

Collection of socioeconomic data was guided by a pre-defined conceptual framework (Fig. 1), hypothesising that: (1) relative agricultural success is associated with higher SEP (Box 1), (2) higher SEP reduces malaria risk and (3) the effect of SEP on malaria risk is mediated by treatment-seeking behaviour, house type and food security among other variables.

### Household and women’s surveys

Socioeconomic data were collected during three surveys: (i) a household survey conducted at baseline, (ii) a second household survey conducted after 24 months of follow-up in September-October 2013 and (iii) a women’s survey, administered as a separate structured questionnaire alongside the second household survey. Both household surveys were administered to one designated adult respondent from each household, if they met four inclusion criteria: (1) usually resident, (2) present in the sampled household the night before the survey, (3) aged at least 18 years and (4) agreed to provide informed written consent. The women’s survey was administered to all adult women of childbearing age (18–49 years), resident in each study household, who met three inclusion criteria: (1) usual female resident, (2) present in the sampled household the night before the survey, (3) agreed to provide informed written consent. Households were excluded if no adult respondent could be located on more than three occasions over two weeks.

Variables for the wealth index were collected in the first household survey (main mode of transport to the health facility) and in the second household survey (all other wealth index variables). House construction was recorded through separate house visits by the entomology field teams during 2013 and validated by the second household survey. Agricultural data were collected in the second household survey. The educational status of each child’s mother or the eldest female caregiver in each child’s household was recorded in the women’s survey.

### Data analysis

Data were collected using standardised record forms entered into Microsoft Access for follow-up of study participants and using a paperless system for the household and women’s surveys.

#### Wealth index and house type

We used a wealth index previously developed for the study population [15]. In brief, principal component analysis (PCA) was used to create the wealth index from nine variables: ownership of (1) mobile telephones, (2) radios, (3) clocks, (4) cupboards, (5) sofas and (6) tables; (7) number of people per sleeping room; (8) access to an improved toilet and (9) main mode of transport to the health facility. Households were ranked by wealth scores and grouped into tertiles to give a categorical measure of SEP. A definition of house type previously developed for the study area was used [16]. Main wall material, main roof material and eave type were used to classify homes as either modern (wood, cement or brick walls; a metal or tiled roof and closed eaves) or traditional (all other homes).

There were four components within the analysis that evaluated: (1) the association between agricultural success and SEP, (2) risk factors for human biting rate (HBR), parasite prevalence and incidence of clinical malaria, including SEP, (3) spatial clustering of socioeconomic variables and (4) mediators of the association between SEP and parasite prevalence.

1. *Association between agricultural success and SEP:* Agricultural success was estimated through household survey questions on indicators within five domains, after Oya [17] and Scoones [18] (Fig. 1): (1) land area cultivated, (2) farm labour, (3) capitalisation (access to advanced means of production, such as pesticides or heavy machinery), (4) productivity and (5) market engagement (proportion of produce sold versus used for own consumption). Cross tabulations and Pearson’s chi-square test were used to explore the associations between indicators of agricultural success, wealth index tertiles and food security.
2. *Risk factors for malaria:* For each risk factor, including SEP, we modelled its association with HBR, parasite prevalence and incidence of clinical malaria. Negative binomial regression was used to model the number of *Anopheles* caught per household per night and the number of malaria cases per child with the number of catch nights and person years included as offset terms. The odds of malaria infection at the time of each routine clinic visit were modelled using logistic regression. For the clinical outcomes (parasite prevalence and malaria incidence), age and gender were included in the model as covariates and robust standard errors were used to adjust for repeat measures (clustering) at the household level.
3. *Spatial analysis of socioeconomic variables:* Spatial autocorrelation (clustering) of three socioeconomic variables (cultivated land area, wealth index scores and house type) was explored at global scale using univariate Moran’s *I* and at local scale using univariate Anselin Moran’s *I* (Additional file 2).
4. *Mediation of the association between SEP and malaria:* We aimed to calculate the effect of SEP on malaria infection risk that is mediated through treatment-seeking behaviour, house type and food security using the algorithm described by Imai [19] (Additional file 3; Fig. 1). This algorithm makes two ignorability assumptions which in practice will hold if there is no unmeasured confounding of the association between exposure and mediator, exposure and outcome or mediator and outcome, and there is no reverse causation.

## Results

### Study population

**Fig. 2:**
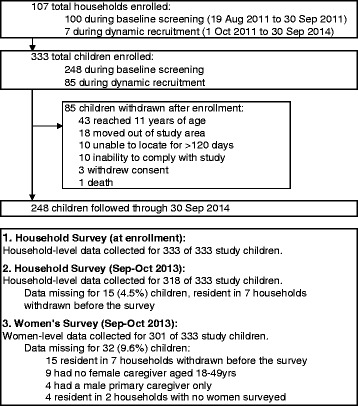
Study profile for a cohort of children aged 6 months to ten years (*N* = 333) in Nagongera, Uganda

A total of 333 children in 107 total households were enrolled between August 2011 and September 2014 (Fig. 2). The mean age of study children during follow-up was 5.7 years and 153 (46 %) were female.

### Wealth index

The first principal component explained 29.3 % of overall variability in the asset variables. The weight assigned to each variable was: cupboard (0.45), clock (0.43), sofa (0.41), table (0.37), mobile (0.30), toilet access (0.29), radio (0.29), people per sleeping room (0.19), mode of transport to health facility (0.10). Wealthier households generally sought treatment for fever faster and had better education, housing and food security than poorer households (Table 1).

**Table1:** Characteristics of study participants and households in Nagongera, Uganda

Characteristic	Wealth index tertile (%)			
	Poorest	Middle	Highest	*P*
Characteristics of children (N = 333)				
Mean age during follow up in years	5.6	5.6	5.8	0.61
Female	41.8	45.8	50.5	0.45
Female caregiver completed at least primary education^a^	7.5	26.0	27.6	0.003
Female caregiver seeks fever treatment on same day^b^	28.8	8.2	42.0	<0.001
Characteristics of households (N = 100)				
Distance to nearest health facility <3 km	54.3	40.6	48.5	0.53
Health expenditure ≥25 % of total household expenditure	8.6	6.3	18.2	0.26
Modern house^c^	0.0	25.0	48.5	<0.001
Meat eaten ≥3 days per week	17.1	37.5	66.7	<0.001
Meals per day ≥3	2.9	28.1	54.6	<0.001
Land area cultivated ≥1.6 ha^d^	28.6	34.4	60.6	0.02

^a^Data on female caregiver’s education collected for 301 of 333 (90 %) children

^b^Data on female caregiver’s treatment-seeking behaviour collected for 191 of 333 (57 %) children

^c^Modern house: Cement, wood or metal wall; tiled or metal roof and closed eaves. Traditional house: all other houses

^d^Ha = hectare; 1.6 ha = 4 acres

### Association between agricultural success and SEP

All households grew crops and agriculture was the primary source of income for 74 % of households. Wealthier households cultivated more land and had greater agricultural income, compared to the lowest tertiles. Wealthier households and those with larger farms also employed more farm labour, were more likely to use an oxplough, owned more tropical livestock units and sold a greater proportion of their crops than poorer households and those with smaller farms (Table 2). Households with larger farms reported fewer problems getting food to eat (*P* = 0.001) and ate meat more frequently (*P* = 0.002).

**Table 2:** Association between agricultural success, land area cultivated and household socioeconomic position in 100 households in Nagongera, Uganda

Indicator	Land area cultivated (%)			Wealth index tertile (%)			
	<1.6 ha^a^ (*N* = 59)	≥1.6 ha (*N* = 41)	*P*	Poorest (*N* = 35)	Middle (*N* = 32)	Highest (N = 33)	*P*
Land area cultivated							
Land area cultivated (≥1.6 ha vs <1.6 ha)^a^	-	-	-	28.6	34.4	60.6	0.02
Land ownership (all owned vs part rented)	35.6	51.2	0.12	45.7	34.4	45.5	0.57
Farm labour							
Hired farm labour	50.9	61.0	0.32	42.9	43.8	78.8	0.004
Total number of farm workers (≥6 people vs 0–5 people)	25.4	51.2	0.008	17.1	31.3	60.6	0.001
Capitalisation and inputs							
Ox-plough used, past 12 months	33.9	73.2	<0.001	34.3	40.6	75.8	0.001
Pesticides and herbicides used, past 12 months	69.5	78.1	0.34	65.7	75.0	78.8	0.46
Access to credit for agriculture	15.3	29.3	0.09	17.1	18.8	27.3	0.55
Productivity							
TLU^b^ per household member (≥0.05 vs <0.05 TLU per person)	33.9	61.0	0.007	37.1	34.4	63.6	0.03
Market engagement							
Total income from crop sales, past 12 months^c^	27.1	51.2	0.002	20.0	31.3	60.6	0.01
Total income from crop and livestock sales, past 12 months^d^	18.6	40.0	0.001	11.4	18.8	53.1	0.001
Proportion of crops sold (≥25 % vs <25 %)	22.0	48.8	0.005	17.1	31.3	51.5	0.01
Non-agricultural income							
Main source of household income^e^	-	-	-	11.4	15.6	21.2	0.27
Remittances received, past 12 months	-	-	-	5.7	12.5	27.3	0.04

^a^Ha = hectare; 1.6 ha = 4 acres

^b^Tropical Livestock Units (TLUs) are a standardised method for quantifying livestock. One TLU corresponds approximately to 250 kg animal weight and total TLUs are calculated by assigning region-specific weights to different livestock types. The following weights were assigned, after Chilonda and Otte: 0.5 per cattle, 0.1 per goat, 0.01 per poultry or rabbit [32]

^c^Total income from all crop sales in the past 12 months: ≥US$ 80 versus < US$ 80 (2013 prices)

^d^Total income from crop and livestock sales in the past 12 months: ≥US$ 120 versus < US$ 120 (2013 prices)

^e^Main source of household income: skilled labour versus remittances, agriculture or manual labour

### Risk factors for malaria

#### Human biting rate (HBR)

A total of 124,746 adult female *Anopheles* were caught over 3489 collection nights, yielding an overall HBR of 35.8 *Anopheles* per house per night. HBR was 29 % lower in the wealthiest households (highest versus lowest wealth index tertile: Incidence Rate Ratio (IRR) 0.71, 95 % confidence intervals (*CI*) 0.54–0.93, *P* = 0.01) and 47 % lower in households with good house construction, controlling for household SEP (modern versus traditional housing: adjusted IRR 0.53, 95 % *CI* 0.40–0.69, *P* < 0.001) (Table 3).

**Table 3:** Socioeconomic risk factors for human biting rate in 100 households in Nagongera, Uganda

Characteristic		HBR (Total collection nights)^a^	IRR (95 % *CI*)^b^	*P*
Wealth index tertile	Poorest	41.5 (1136)	1	0.01
	Middle	34.4 (1132)	0.86 (0.65–1.13)	
	Highest	28.8 (1110)	0.71 (0.54–0.93)	
House type^c^	Traditional	40.5 (2690)	1	<0.001
	Modern^d^	19.9 (799)	0.53 (0.40–0.69)	

^a^HBR: Human biting rate: total adult female *Anopheles* caught/total collection nights

^b^IRR: Incidence rate ratio; CI: Confidence interval

^c^IRR for this variable was adjusted for household wealth

^d^Modern house: Cement, wood or metal wall; tiled or metal roof and closed eaves. Traditional house: all other houses

#### Malaria infection

A total of 3367 routine blood smears were taken of which 1037 (30.8 %) were positive. All participants contributed at least one smear. Controlling for age and gender, the odds of infection were 49 % lower in children living in modern housing (modern versus traditional housing: adjusted Odds Ratio (*OR*) 0.51, 95 % *CI* 0.36–0.71, *P* < 0.001), 48 % lower in wealthier children (highest versus lowest wealth index tertile: adjusted *OR* 0.52, 95 % *CI* 0.35–0.78, *P* = 0.001) and 36 % lower in children with good food security (meat eaten 3–7 versus 0–2 days per week: adjusted *OR* 0.64, 95 % *CI* 0.47–0.88, *P* = 0.007) (Table 4).

**Table 4:** Socioeconomic risk factors for malaria in children aged six months to 10 years in Nagongera, Uganda

Characteristic		Malaria infection			Incidence of clinical malaria		
		PR (Total blood smears)^a^	OR (95 % *CI*)^b^	*P*	Malaria incidence (total person years)^c^	IRR (95 % *CI*)^d^	*P*
Mean age during follow-up	6 m to <3 years	19.2 (657)	1	<0.001	4.1 (134)	1	<0.001
	3 to <5 years	27.6 (699)	1.60 (1.18–2.18)		4.2 (177)	1.01 (0.85–1.19)	
	5 to <11 year	35.7 (2011)	2.34 (1.77–3.09)		2.3 (491)	0.54 (0.46–0.65)	
Gender	Female	29.9 (1518)	1	0.54	2.7 (361)	1	0.12
	Male	31.5 (1849)	1.07 (0.86–1.35)		3.2 (441)	1.13 (0.97–1.32)	
Wealth index tertile	Lowest	38.4 (1087)	1	0.001	3.0 (258)	1	0.66
	Middle	29.6 (1170)	0.65 (0.48–0.87)		3.1 (280)	1.12 (0.90–1.40)	
	Highest	25.3 (1010)	0.52 (0.35–0.78)		2.9 (241)	1.05 (0.83–1.34)	
Female caregiver’s level of education	None	33.4 (788)	1	0.21	3.5 (188)	1	0.005
	Incomplete 1^ry^	31.7 (1703)	0.96 (0.68–1.36)		3.0 (406)	0.83 (0.69–1.01)	
	1^ry^ or higher	26.6 (593)	0.74 (0.48–1.15)		2.4 (140)	0.69 (0.53–0.91)	
Distance to health facility	3–6 km	33.4 (1994)	1	0.07	2.9 (474)	1	0.56
	0–2 km	27.1 (1373)	0.75 (0.55–1.02)		3.1 (328)	1.06 (0.87–1.29)	
Time for female caregiver to seek treatment for fever	≥1 day	29.5 (1434)	1	0.55	3.3 (342)	1	0.31
	Same day	27.5 (509)	0.86 (0.51–1.42)		2.5 (120)	0.87 (0.67–1.13)	
Proportion of household expenditure on health	<25 %	31.0 (3059)	1	0.65	3.1 (730)	1	0.15
	25–50 %	34.1 (208)	1.15 (0.63–2.10)		2.0 (49)	0.73 (0.48–1.12)	
House type^e^	Traditional	32.9 (2794)	1	<0.001	3.0 (665)	1	0.67
	Modern	20.4 (573)	0.51 (0.36–0.71)		2.7 (136)	0.93 (0.68–1.28)	
People per sleeping room	>2 people	31.9 (2752)	1	0.24	3.1 (656)	1	0.29
	0–2 people	27.0 (515)	0.78 (0.51–1.19)		2.6 (123)	0.86 (0.64, 1.14)	
Days eating meat per week	0–2 days	34.6 (2123)	1	0.007	3.0 (507)	1	0.71
	3–7 days	24.7 (1144)	0.64 (0.47–0.88)		2.9 (271)	0.96 (0.77–1.20)	
Meals per day	2 meals	33.1 (2439)	1	0.05	3.0 (581)	1	0.78
	3–4 meals	25.6 (828)	0.72 (0.52–1.00)		2.9 (197)	0.96 (0.75–1.24)	

^a^PR: *Plasmodium falciparum* parasite rate: total positive blood smears/total blood smears

^b^OR: Odds Ratio adjusted for age at the time of the blood smear and gender. *CI* Confidence interval

^c^Malaria incidence per person year: total malaria episodes/total person years at risk

^d^IRR: Incidence Rate Ratio adjusted for mean age during follow-up and gender

^e^Modern house: Cement, wood or metal wall; tiled or metal roof and closed eaves. Traditional house: all other houses

#### Incidence of clinical malaria

A total of 2399 episodes of uncomplicated malaria were diagnosed after 802 person years of follow-up, yielding an overall incidence of 3.0 episodes per person year at risk. One participant was withdrawn immediately after enrolment without contributing person time. Controlling for age and gender, malaria incidence was 31 % lower among children with better-educated female caregivers (completed at least primary versus no education: adjusted IRR 0.69, 95 % *CI* 0.53–0.91, *P* = 0.008). Malaria incidence was not associated with any other risk factors explored (Table 4).

### Spatial analysis of socioeconomic variables

**Fig. 3:**
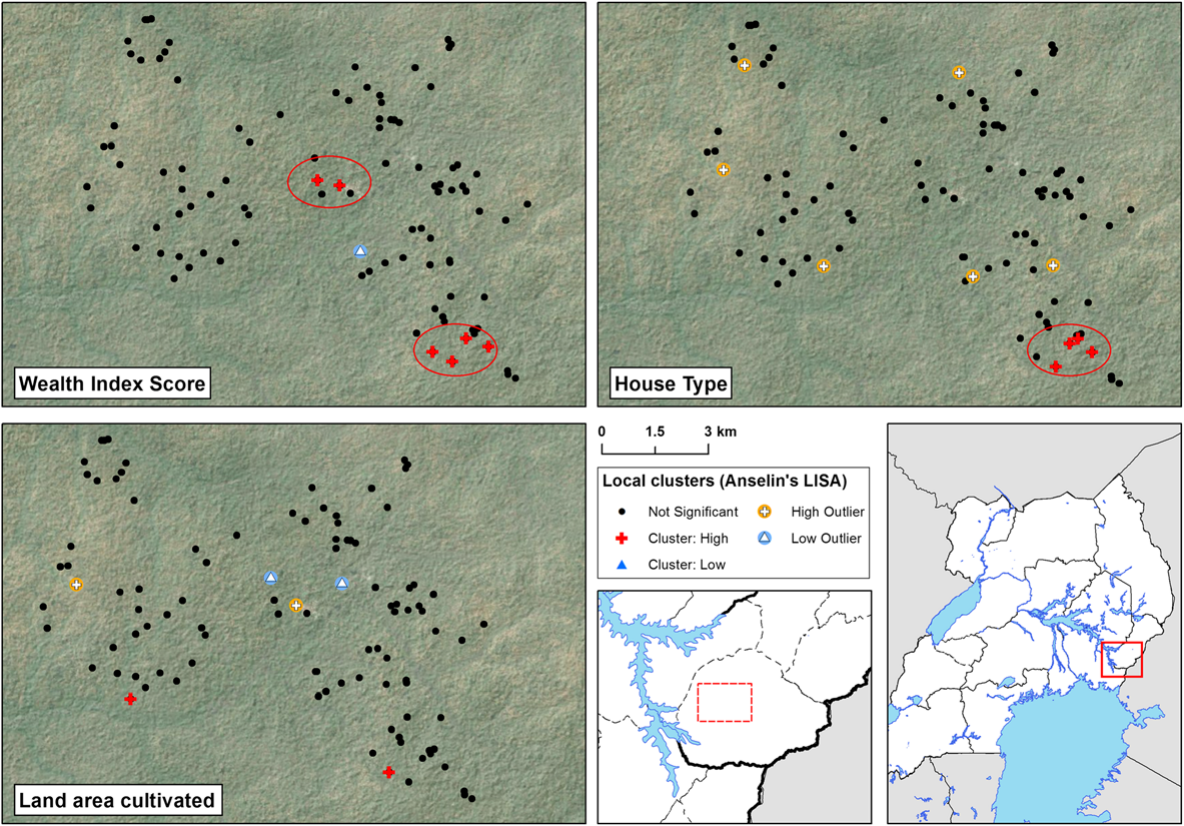
Local cluster maps of wealth index score, house type and cultivated land area in 100 households in Nagongera, Uganda. Maps show results from univariate Local Indicator of Spatial Association (LISA) analysis. A cluster of high wealth index scores overlapping with a cluster of modern housing is located in the south-east of the study area. Houses were classified as modern (cement, wood or metal walls; a tiled or metal roof and closed eaves) or traditional (all other houses). Wealth index score and land area cultivated were modelled as continuous variables

Across the whole study area, there was no evidence of clustering of cultivated land area, house type or wealth index (Additional file 2). However, there was local clustering of these three variables, with a cluster of modern housing and high wealth index scores in study houses located in a small town (Nagongera) in the south east of the study area (Fig. 3).

### Mediation of the association between SEP and malaria

There was evidence that the total effect of SEP on malaria infection risk in children was partly explained by differences in house quality (24.9 %, 95 % *CI* 15.8 % – 58.6 %) and food security (18.6 %, 95 % *CI* 11.6 % – 48.3 %) between wealthier and poorer homes. Treatment-seeking behaviour was excluded from the mediation analysis since data on time to seek treatment were available for 191 (57 %) children only (Table 5).

**Table 5:** Mediation analysis of the association between socioeconomic position and malaria infection in children aged six months to 10 years in Nagongera, Uganda

Mediating variable^a^	Risk difference (95 % CI)^b^, high versus low SEP^c^			Proportion of total effect of SEP that occurs through mediator, % (95 % *CI*)
	Direct effect of SEP	Effect of SEP through mediator	Total effect of SEP	
House type^d^	−8.6 (-15.6, -2.1)	−2.9 (-5.5, -0.8)	−11.5 (-18.1, -4.9)	24.9 (15.8, 58.6)
Food security^e^	−9.2 (-16.9, -2.2)	−2.1 (-5.3, 0.0)	−11.4 (-18.4, -4.4)	18.6 (11.6, 48.3)

^a^Treatment-seeking behaviour was excluded from the mediation analysis since data on time to seek treatment were available for 191 of 333 (57 %) children only

^b^Risk difference adjusted for gender, age (<5 years vs 5–11 year) and clustering at the household level

^c^SEP: household socioeconomic position, modelled as a binary variable (middle and highest wealth index tertiles versus lowest wealth index tertile)

^d^House type: modern (cement, wood or metal walls; and tiled or metal roof; and closed eaves) versus traditional (all other houses)

^e^Food security: Meat consumed 3–7 days versus 0–2 days per week

## Discussion

We investigated the association between socioeconomic position (SEP), its determinants, and malaria in children in a rural, high-transmission setting in Uganda. Households with greater agricultural success had higher SEP. In turn, households and children of higher SEP were exposed to a 29 % lower HBR and had 48 % lower odds of malaria infection than the poorest. Finally, there was evidence that the association between SEP and malaria infection was explained partly by house type and food security. Our findings concur with observations elsewhere in sub-Saharan Africa (SSA) that the odds of malaria infection are on average doubled in children with the lowest SEP (as measured by household wealth index scores or parent’s educational status or occupation) compared to children with the highest SEP within the same community [3]. Socioeconomic factors may be as influencial in malaria transmission today in Uganda as they were historically in North America and Europe [4].

To our knowledge, the present study is the first to use mediation analysis to explore the causal pathways by which poverty may cause malaria. First, the analysis suggests that house type may explain part of the association between SEP and malaria infection risk, consistent with previous observations that well-built housing, with closed eaves and modern wall and roof materials, is associated with lower malaria risk through reduced mosquito house entry [11, 16]. Second, we observed that food security may also mediate the poverty-malaria association. While findings on the relationship between nutrition and malaria are inconsistent [20], there is evidence that undernutrition may be associated with greater susceptibility to malaria infection and progression to severe disease [10] and that protein-energy malnutrition is associated with greater malaria morbidity and mortality [21]. Indeed, a previous study in our study district found that stunting (an indicator of chronic malnutrition) was associated with a higher incidence of clinical malaria in children [22]. Conversely, it is possible that our measure of food security was more of a proxy for SEP than nutritional status [23].

Identifying factors potentially mediating between SEP and malaria provides evidence of a biologically plausible mechanism for causality, yet the mediation analysis was subject to a number of limitations. First, house quality and food security together accounted for less than half of the association between poverty and malaria infection risk, suggesting that other mediators remain unaccounted for. While treatment-seeking behaviour was excluded from the mediation analysis, wealthier households sought treatment for fever more promptly than poorer households, so this variable merits future evaluation as a potential mediator. Additional potential mediators may include distance of households to the village periphery, housing density and, given the local clustering of wealthier households, malaria risk in neighbouring households. Education level, while considered an indicator of SEP [15], arguably could also lie on the mediation pathway. Therefore our conceptual framework and analysis were not exhaustive and provide only a preliminary exploration of the complex relationships linking poverty and malaria. Second, the assumptions underlying the mediation analysis may not have been fully met. For example, the costs of malaria can worsen poverty, resulting in reverse causality [7, 8], and the relationship between SEP and malaria may be confounded by environmental factors such as distance to larval habitats (alternatively, location might be on the causal pathway between SEP and malaria). While we aimed to omit from the wealth index variables directly associated with malaria [15], some of the included assets may have been associated with both SEP and house type (e.g. sofa ownership or toilet access). Third, we did not observe any association between SEP and incidence of clinical malaria and the interpretation of this finding is unclear.

To identify potential cross-over between development interventions and malaria control, we sought to understand better the heterogeneity in SEP in the study area. Overall we found that SEP was associated with increased odds of malaria infection. In turn, SEP was associated with relative agricultural success, consistent with agriculture being a major livelihood source in Nagongera as in much of rural Africa [18, 24]. We also observed that wealthier households had larger farms and were overall more successful in agriculture than poorer households. Of course, wealthier households may invest more in agriculture and other enterprises, improving their overall productivity. Yet it is also feasible that agricultural productivity limits household wealth and that land access constrains productivity in Nagongera, since there is extensive land fragmentation stemming from the division of land over generations, which is likely to continue as the Ugandan population expands from 39 million in 2015 to an estimated 102 million in 2050 [25]. Elsewhere in SSA, rural poverty has been linked to lower vegetation index scores, remoteness and poor soil fertility [26]. While the conclusions that may be drawn from our observational study are limited, our findings highlight the importance of understanding malaria transmission within the wider social and ecological landscape.

By examining the relationship between poverty and malaria, practical steps towards multisectoral intervention may be identified. First, there may be overlap between poverty reduction and malaria control [3]. If this is the case, interventions such as Farmer Field Schools (a group-based education approach) might be targeted in areas where agriculture is an important livelihood source to increase production and marketing capacity while incorporating training in Integrated Pest and Vector Management [27]. If land access constrains productivity, diversification into non-agricultural activities may be necessary, alongside interventions to improve productivity and market access among remaining farmers. Second, since house quality is associated with malaria risk, malaria control progammes could work with other sectors to scale-up ‘healthy’ housing [28]. Possible strategies may include microfinance initiatives, education and the use of model houses to encourage good house design, or collaboration with other ministries and the private sector [29]. Third, should good nutrition be protective against malaria, nutrition-sensistive interventions – including those related to agriculture and food security – may be complementary to malaria control.

Our study has a number of limitations. First, the mediation analysis was based on untestable assumptions (Additional file 3). Should these assumptions not hold, this would limit confidence in house quality and food security being mediators of the SEP-malaria relationship and in their associated mediating effects. Throughout our analysis, we assume that SEP affects malaria risk, yet reverse causality from malaria risk to SEP and agricultural productivity is highly probable [7, 8, 30]. Second, the conceptual framework was not an exhaustive representation and we were unable to investigate all causal pathways linking SEP and malaria, nor all potential determinants of poverty. Third, the wealth index is an imperfect metric and its representation of underlying SEP is influenced by the variables included in the index [15]. Fourth, our spatial analysis modelled few variables relevant to malaria. Finally, we studied only one population at one time point, so the findings require future validation in this and other settings. Despite the methodological challenges, it is hoped that our analysis offers a preliminary insight into the complex relationship between poverty and malaria, providing a framework for future interdisciplinary research.

## Conclusions

Housing improvements and agricultural development interventions to reduce poverty merit further investigation as multisectoral interventions against malaria. Further interdisplinary research is needed to understand fully the complex pathways between poverty and malaria and to develop strategies for sustainable malaria control.

## **Box 1. Understanding poverty reduction in rural Uganda**

In low income countries, poverty reduction generally involves decreased livelihood vulnerability, changes in livelihood activities and increased incomes through a shift towards more productive activities [24]. In rural areas, such activities are typically grounded in agriculture, later diversifying to include non-agricultural activities [17]. Indeed, agriculture is the primary source of livelihood for much of the rural poor in developing countries. In Uganda, the net output of agriculture comprised 24 % of gross domestic product and the agricultural sector provided two-thirds of total employment in 2010 [31]. In much of rural Africa, heterogeneity in socioeconomic position (SEP) can therefore be understood by examining relative success in smallholder agriculture [17]. Often mistaken as homogeneous, rural African populations encompass many classes with different ambitions and constraints. Relative agricultural success reflects the degree to which smallholder farmers successfully derive a living from the land, sometimes using agricultural income to upscale other enterprises. Agricultural success can be approximated using indicators such as farm size, production performance (yield) and labour hire, or by examining the processes of accumulation and production that allow people to ‘hang in’ (maintain livelihood levels through farming), ‘step up’ (invest in assets to expand current activities, increase production and improve livelihoods), or to accumulate resources to ‘step out’ (move into different activities with higher returns) [18, 24].

## Abbreviations

CI, confidence interval; HBR, human biting rate; IRR, incidence rate ratio; LISA, Local Indicator of Spatial Association; LLIN, long-lasting insecticide-treated bednet; OR, odds ratio; PCA, principal component analysis; SEP, socioeconomic position; SSA, Sub-Saharan Africa; USA, United States of America

## Additional files

Additional file 1: Multilingual abstracts in the six official working languages of the United nations. (PDF 823 kb) Additional file 2: Implementation of the spatial autocorrelation analysis. (PDF 857 kb) Additional file 3: Implementation of the mediation analysis. (PDF 280 kb) Additional file 4: Data files. (ZIP 15 kb)
