# Supplementary material for: Why is malaria associated with poverty? Findings from a cohort study in rural Uganda
Source: Infect Dis Poverty. 2016 Aug 4;5:78. doi: 10.1186/s40249-016-0164-3 (PMC4972958; doi:10.1186/s40249-016-0164-3)
Supplement: Additional file 1: — Multilingual abstracts in the six official working languages of the United nations. (PDF 823 kb) [file 40249_2016_164_MOESM1_ESM.pdf]

Translation of the abstract into the six official working languages of the United Nations

**لم يتم الربط بين مرض الملاريا والفقر؟ نتائج دراسة شملت عينة من المناطق الريفية في أوغندا**

لوسي اس تاستينغ- جون ريك- ايمانيويل ارينيتوي- سارة ستادك- موسى كاميا- جورج كانو- كريستيان بوتتوملي- ديبورا جونستون- جرانت دورسي- ستيف ليندسي- جو لاينز.

Lucy S. Tusting, John C. Rek, Emmanuel Arinaitwe, Sarah G. Staedke, Moses R. Kamya, Jorge Cano, Christian Bottomley, Deborah Johnston, Grant Dorsey, Steve W. Lindsay, Jo Lines

**ملخص الدراسة**

**خلفية الموضوع**: هناك ارتباط بين مرض الملاريا والتنمية المستدامة الا ان تنفيذ تدخل "متعدد القطاعات" مقيد بسبب الفهم المحدود للعلاقة المتلازمة بين الملاريا والفقر. لقد تمت دراسة العلاقة بين الحالة الاجتماعية والاقتصادية و والمسببات المحتملة لها ونسبة الاصابة بمرض الملاريا في ناغونغرا (Nagongera) في ريف اوغندا.

**منهجية البحث**: تم جمع معلومات عن الحالة الاجتماعية والاقتصادية ل 318 طفل تتراوح أعمارهم بين ستة أشهر وعشر سنوات شملت مئة أسرة. وتمت متابعة حالة هذه العينة لمدة 36 شهرا. وسجلت نسبة البعوض بستخدام المصيدة الضوئية للبعوض. وقد تم قياس انتشار الطفيليات بشكل روتيني كل ثلاث أشهر وتم تحديد حالات الاصابة بمرض الملاريا من خلال الحالات السلبية. أولا: قمنا بتقييم العلاقة بين نجاح القائمين على المشاريع الزراعية الصغيرة (حيث ان هذه المشاريع هي المصدر الرئيسي للرزق) و الحالة الاجتماعية والاقتصادية. ثانيا: استكشفنا عوامل الخطورة بين نسبة لدغة البعوضة للبشر و انتشار الطفيليات وحالات الاصابة بمرض الملاريا و التجمع للمتغيرات الاجتماعية و الاقتصادية. ثالثا: درسنا الدور الذي قد تلعبه العوامل المختارة في الربط بين الحالة الاقتصادية والاجتماعية و الاصابة بمرض الملاريا.

**النتائج**: ارتبط النجاح النسبي في المجال الزراعي بنسب تحسن الوضع الاجتماعي والاقتصادي. بالمقابل ارتبط تحسن الوضع الاجتماعي والاقتصادي بانخفاض نسبة لسعات البعوض (النسب الأعلى مقابل النسب الأدنى لمؤشرات الثروة: نسبة معدل الاصابة 0.71, 95% فواصل الثقة 0.93-0.54, p=0.01) وانخفاض احتمالات الأصابة لدى الأطفال (النسب الأعلى مقابل النسب الأدنى لممؤشرات الثروة: نسبة الاحتمال المعدلة 0.52, 95% CI 0.35-0.78, P=0.001). الا ان الوضع الاجتماعي والاقتصادي لم يكن مرتبط بنسب الاصابة السريرية بمرض الملاريا. وأظهر تحليل الوسيط أن جزء من الأثر العام للوضع الاجتماعي والاقتصادي له أثر على خطورة الاصابة بمرض الملاريا والتي قد ترتبط بنوع المنزل (24.9%, 95% CI 15.8–58.6%) والأمن الغذائي بنسبة (18.6%, 95% CI 11.6–48.3%). و لم يتم تحقيق فرضية دور تحليل الوسيط في هذه الدراسة بشكل كامل.

**الخاتمة**: تعد تحسينات الإسكان والتدخل لتطوير التنمية الزراعية لتقليل نسب الفقر ميزة للمزيد من البحث حول التدخل متعدد القطاعات لمكافحة مرض الملاريا. هناك حاجة إلى المزيد من الأبحاث لفهم العلاقة المتلازمة والمعقدة بين الفقر والاصابة بمرض الملاريا بشكل كامل ووضع استراتيجيات للسيطرة المستدامة على انتشار مرض الملاريا.

Translated from English version into Arabic by Randa82, through

TRANSLATORS WITHOUT BORDERS

**为什么疟疾与贫困相关？来自乌干达农村地区队列研究的发现**

Lucy S. Tusting, John C. Rek, Emmanuel Arinaitwe, Sarah G. Staedke, Moses R. Kamya, Jorge Cano, Christian Bottomley, Deborah Johnston, Grant Dorsey, Steve W. Lindsay, Jo Lines

**摘要**

**引言：**疟疾防治与可持续发展密切相关，但是由于对贫困与疟疾之间因果途径的认识有限，“多部门”干预措施的施行受到了限制。我们研究了乌干达农村托罗罗区社会经济地位、社会经济地位的潜在决定因素和疟疾三者之间的关系。

**方法：**我们搜集了 100 个家庭 6 个月到 10 岁的 318 个儿童的社会经济信息，并对这些儿童进行了 36 个月的追踪调查。每月通过诱蚊灯捕蚊记录蚊虫密度。每 3 个月常规测定寄生虫患病率，通过被动病例侦查确定疟疾发病率。首先，我们评估了小户农业（主要生活来源）成功与社会经济地位的关系。其次，探究了叮人率、寄生虫患病率和临床疟疾发病率的社会经济风险因素，以及社会经济变量的空间聚集性。最后，考察了选定的因素在介导社会经济地位与疟疾中的作用。
**结果：**相对的农业成功与较高的社会经济地位有关。相反，高的社会经济地位与较低的叮人率（最高与最低财富指数组相比：发病率比率为 0.71, 95%置信区间(*CI*)为 0.54-0.93, *P*=0.01）以及较低的儿童疟疾感染率（最高与最低财富指数组相比：调整后的比值比为 0.52, 95%*CI* 为 0.35-0.78，*P*=0.001）有关，但社会经济地位与临床疟疾发病率无关。中介分析显示，社会经济地位对疟疾感染的影响一部分可由房屋类型（24.9%, 95% *CI* 15.8-58.6%）和食品安全（18.6%, 95% *CI* 11.6-48.3%）来解释；然而，中介分析的假设可能并不完全符合要求。
**结论：**当多部门参与防治疟疾时，需要对减少贫困的住房条件改善和农业发展措施做进一步的调查。为了更全面的认识贫困和疟疾之间复杂的关联途径以及发展可持续的疟疾防治策略，我们需要更深入的进行跨学科研究。

Translated from English version into Chinese by Sun Lei, edited by Yang Pin, through

CHINESE CENTER FOR DISEASE CONTROL AND PREVENTION
NATIONAL INSTITUTE OF PARASITIC DISEASES

**Pour quelle raison le paludisme est-il lié à la pauvreté ? Conclusions d'une étude de cohorte menée en milieu rural en Ouganda**

Lucy S. Tusting, John C. Rek, Emmanuel Arinaitwe, Sarah G. Staedke, Moses R. Kamya, Jorge Cano, Christian Bottomley, Deborah Johnston, Grant Dorsey, Steve W. Lindsay, Jo Lines

**Résumé**

**Historique** : La lutte contre le paludisme et le développement durable sont liés mais la mise en place d'une intervention "multisectorielle" est réduite en raison d'une compréhension insuffisante des liens de causalité entre la pauvreté et le paludisme. Nous avons étudié la relation entre le facteur socio-économique, les données déterminantes de ce facteur et le paludisme à Nagongera, en milieu rural en Ouganda..
**Procédés** : des données socio-économiques furent recueillies sur 318 enfans agés de six mois à 10 ans et vivant au sein de 100 foyers : ces enfants ont été suivis pendant plus de 36 mois. La densité de moustiques fut enregistrée grâce à des collectes de piège à lumière effectuées mensuellement.
La prévalence parasitaire a été mesurée quotidiennement tous les trois mois et l'incidence du paludisme a été déterminée par des données de détection passive. En premier lieu, nous avons évalué le lien entre les petites exploitations agricoles florissantes (principal moyen de subsistance) et les facteurs socio-économique. En deuxième lieu, nous avons étudié les facteurs de risque socio-économiques du taux d'infection chez l'homme, de la prévalence parasitaire, de l'impact du paludisme clinique et des agrégats spatiaux des facteurs socio-économiques. En troisième lieu, nous avons étudié le rôle des facteurs sélectionnés en modulant le lien entre le facteur socio-économique et le paludisme.
**Résultats:** le succès relatif des exploitations agricoles a été lié avec un facteur socio-économique plus important. A son tour, un facteur socio- économique plus élevé a été lié avec un taux d'infection du paludisme plus bas (tertiel de l'indicateur de richesse le plus grand *versus* le plus bas) ratio du taux d'incidence 0 71, 95¨% d'intervalles de confiance (CI) 0.54-0.93, p=0.01) et des pourcentages plus bas de l'infection du paludisme chez les enfants (tertiel d'indicateur de richesse le plus haut *versus* le plus bas) ratio du taux d'incidence corrigé 0 52 (95% CI 0.35-0.78, p=0.001), mais le facteur socio-économique

n'a pas été lié avec l'impact du paludisme clinique. L'analyse intermédiaire a montré qu'une partie de l'impact total du facteur socio-économique sur le risque d'infection du paludisme était expliquée par le type d'habitation (24.9%, 95% CI 15.8–58.6) et par la sécurité alimentaire 18.6%, 95% CI 11.6–48.3%) ; cependant les hypothèses de l'analyse intermédiaire peuvent ne pas avoir été entièrement vérifiées.

**Conclusions** : Les améliorations des habitations et les actions en matière du développement agricole méritent une enquête approfondie comme des interventions multisectorielles pour lutter contre le paludisme. Une recherche interdisciplinaire approfondie est nécessaire pour bien comprendre les liens complexes de causalité entre la pauvreté et le paludisme et pour développer des stratégies pour lutter contre le paludisme de façon durable.

Translated from English version into French by veromarie, through

TRANSLATORS
WITHOUT BORDERS

**Почему малярия ассоциируется с бедностью? Выводы группового исследования в сельских районах Уганды**

Люси С. Тастинг, Джон С. Рек, Эммануэл Аринаитве, Сара Дж. Стедке, Мозес Р. Камья, Йорге Кано, Кристиан Боттомли, Дебора Джонсон, Грант Дорси, Стив В. Линдси, Джо Лайнс

**Резюме**

**Подоплека:** Борьба с малярией и устойчивое развитие связаны друг с другом, однако реализация «многоотраслевого» вмешательства сдерживается ограниченным пониманием причинно-следственных связей между бедностью и малярией. Мы изучили взаимосвязь между социально-экономическим положением (СЭП), потенциальными факторами СЭП и малярией в Нагонгера, сельской Уганде.

**Методы:** Бал произведен сбор социально-экономической информации о 318 детях в возрасте от 6 месяцев до 10 лет, проживающих в 100 домашних хозяйствах, наблюдение велось 36 месяцев. Плотность комаров фиксировалась ежемесячно при помощи световой ловушки. Распространенность паразитов измерялась регулярно каждые тримесяца и уровень заболеваемости малярией определялся пассивным выявлением больных. Во-первых, мы оценивали связь между успешностью в мелком фермерстве (основной источник существования) и СЭП. Во-вторых, мы изучили социально-экономические факторы риска доли кровососущих, распространения паразита и уровня заболеваемости клинической малярией, а также пространственную кластеризацию социально-экономических переменных. В-третьих, мы исследовали роль выбранных факторов в опосредовании связи между СЭП и малярией.

**Результаты:** Относительный сельскохозяйственный успех был связан с более высокими СЭП. В свою очередь, высокое СЭП сентября было связано с более низкими HBR (самый высокий по сравнению с самым низким тертилем индекса богатства: Коэффициент частоты заболеваний 0,71, 95% доверительный интервал (ДИ) 0.54-0.93, p=0,01) и более низкие шансы малярийной инфекции у детей (самый высокий по сравнению с самым низким тертилем индекса богатства: скорректированное отношение шансов 0.52, 95% ДИ 0.35-0.78, p=0,001), но СЭП не было связано с клинической заболеваемостью малярией. Анализ посредничества предположил, что часть общего влияния СЭП на риск заражения малярией объясняется типом дома (24,9%, 95% ДИ 15.8-58.6%) и продовольственной безопасностью (18,6%, 95% ДИ 11.6-48.3%); однако прогноз анализа посредничества, возможно, оправдался не полностью.

**Заключение:** Улучшение жилищных условий и сельского хозяйства с целью снижения бедности заслуживает дальнейшего исследования в рамках многоотраслевых мер по борьбе с малярией. Необходимы дальнейшие интердисциплинарные исследования для полного понимания сложных связей между бедностью и малярией, и разработки стратегии устойчивой борьбы с малярией.

Translated from English version into Russian by Jekaterina Merkuljeva, through

**Por qué la malaria está asociada a la pobreza? Conclusiones de un estudio de cohorte en Uganda rural**

*Lucy S. Tusting, John C. Rek, Emmanuel Arinaitwe, Sarah G. Staedke, Moses R. Kamya, Jorge Cano, Christian Bottomley, Deborah Johnston, Grant Dorsey, Steve W. Lindsay, Jo Lines*

**Resumen**

**Antecedentes:** El combate a la malaria y el desarrollo sostenible están relacionados, pero la instrumentación de una intervención “multisectorial” se ve limitada porque no se comprende debidamente la relación causal entre la pobreza y dicha enfermedad. Investigamos la relación entre la situación socioeconómica (SSE), los factores potencialmente determinantes de la SSE y la malaria en Nagongera, una población rural de Uganda.

**Métodos:** Se reunieron los datos socioeconómicos de 318 niños de entre 6 meses y 10 años de edad residentes en 100 hogares, a quienes se les hizo seguimiento por un período de hasta 36 meses. Se registró la densidad de mosquitos por medio de trampas livianas. La prevalencia del parásito se midió regularmente cada tres meses, y se determinó la incidencia de la malaria por medio de la detección pasiva de casos. En primer lugar, evaluamos la relación entre la agricultura artesanal (principal fuente de sustento) y la situación socioeconómica. En segundo lugar, exploramos los factores de riesgo socioeconómico asociados al índice de picadura hombre (IPH), a la prevalencia del parásito e incidencia clínica de la malaria, y a la concentración espacial de las variables socioeconómicas. En tercer lugar, investigamos el papel que desempeñan una serie de factores específicos en mediar la asociación de la SSE y la enfermedad.

**Resultados:** El éxito relativo en la agricultura se asoció a una mejor situación socioeconómica. A su vez, una buena SSE se asoció con un menor IPH (tertil de índice de riqueza más alto *versus* tertil de índice de riqueza más bajo: ratio de índice de incidencia 0,71, 95% de intervalos de confianza (IC) 0,54 – 0,93, p = 0,01) y menor probabilidad de contraer la malaria en los niños (tertil de índice de riqueza más alto *versus* tertil de índice de riqueza más bajo: ratio de probabilidad ajustado 0,52, 95% IC 0,35 – 0,78, p = 0,001), pero la SSE no se asoció con la incidencia clínica de la malaria. Del análisis de los factores mediadores de la enfermedad surge que la influencia de la situación socioeconómica en el riesgo de contraer malaria se explica en parte por el tipo de vivienda (24,9%, 95% IC 15,8 – 58,6%) y la seguridad alimentaria (18,6%, 95% IC 11,6 – 48,3%); sin embargo, puede que no se haya probado en su totalidad la hipótesis del análisis de la mediación.

**Conclusión:** Las intervenciones tendientes a mejorar las viviendas y promover el desarrollo de la agricultura para reducir la pobreza ameritan que se profundice la investigación como intervención multisectorial de lucha contra la enfermedad. Se requiere más investigación interdisciplinaria para llegar a comprender cabalmente las complejas relaciones entre la pobreza y la malaria, y desarrollar estrategias para combatir la enfermedad de manera sostenida.

Translated from English version into Spanish by Mónica Algazi, through
