# Supplementary material for: Why is malaria associated with poverty? Findings from a cohort study in rural Uganda
Source: Infect Dis Poverty. 2016 Aug 4;5:78. doi: 10.1186/s40249-016-0164-3 (PMC4972958; doi:10.1186/s40249-016-0164-3)
Supplement: Additional file 2: — Implementation of the spatial autocorrelation analysis. (PDF 857 kb) [file 40249_2016_164_MOESM2_ESM.pdf]

**Additional File 1. Implementation of the spatial autocorrelation analysis**

Spatial autocorrelation (clustering) of three socioeconomic variables (cultivated land area, wealth index scores and house type) was explored at global scale using univariate Moran's *I* [1] and at local scale using univariate Anselin Moran's *I* [2]. The global Moran's *I* estimates the degree of correlation between neighbours over the whole study area, whereas Anselin Moran's *I* measures local clustering [2].

Spatial autocorrelation statistics depend on the definition of neighbourhood relationships through which the spatial configuration of the sampled subpopulation was defined prior to analysis. Delaunay triangulation was used to set up a neighbourhood matrix of sampling units (households). This method is commonly applied to construct neighbours on point features by creating Voronoi triangles [3]. A mesh of non-overlapping triangles is created from feature centroids; features associated with triangle nodes that share edges are neighbours (Figure A1). The sum of weights for a given distance class decreases for large distance classes, and a bias may arise from the fact that only observations at the edge of the sampled population can contribute to the estimates for larger distances. We therefore limited the description of the spatial structure to half the maximum distance between households (around 7.3 km for the study area) [4]. We also standardized the spatial weights so that all weights summed to unity within a group of neighbours (row standardization). The estimate of spatial autocorrelation can be biased when the data are not normally distributed [5]. Accordingly, cultivated area cultivated was transformed by a cubic root function to approach a Gaussian distribution.

*Cluster analysis*: Moran's *I*[1] was used to account for the global spatial autocorrelation of socioeconomic variables. For the Moran's *I* statistic, the sum of covariations between the sites for the distance *d(i,j)* was divided by the overall number of sites $W(d_{i,j})$ within the distance class *d(i,j)*. Thus, the spatial autocorrelation coefficient for a distance class *d(i,j)* was the average value of spatial autocorrelation at that distance.

$$I = \frac{\mathrm{n}}{\mathrm{S}_p} \frac{\sum_{i=1}^{n}\sum_{j=1}^{n} W_{ij}(\gamma_i-\bar{\gamma})(\gamma_j-\bar{\gamma})}{\sum_{i=1}^{n}(\gamma_i-\bar{\gamma})^2}, \text{ where}$$

$n$ = the sample size

$W_{ij} = \begin{cases} 1 \text{ if sites i, j are neighbours} \\ 0 \text{ otherwise} \end{cases}$ = row-standardized spatial weights matrix of sites *i* and *j*

$\mathrm{S}_p = \sum_{i=1}^{n}\sum_{j=1}^{n} W_{i.j}$ = sum of the number of sampling locations per distance class,

$\gamma_i$ = the value at household $i$; $\bar{\gamma}$ = global mean value

The actual value for Moran's *I* was then compared with the expected value under the assumption of complete randomisation.

$$E(I) = -\frac{1}{n-1}$$

Moran's *I* values may range from -1 (disperse) to +1 (clustered). A Moran's *I* value of 0 suggests complete spatial randomness. To verify that the value of Moran's *I* was significantly different from the expected value, a Monte Carlo randomisation test was applied with 9,999 permutations to achieve highly significant values. This statistic is a global statistic in that it averages all cross

outcomes over the entire domain. A local version, called Local Indicator of Spatial Association (LISA) or Anselin Local Moran's *I*[2] allows us to test for statistically significant local spatial clusters, including the type and location of these clusters. It is calculated as follows:

$$I_i(d) = \frac{(\gamma_i - \bar{\gamma})}{\frac{1}{n}\sum_{i=1}^{n}(\gamma_i - \bar{\gamma})} \sum_{i=1}^{n} W_{ij}(d)(\gamma_i - \bar{\gamma}), \text{ where}$$

$W_{ij}(d)$ is the row-standardized weights matrix given a local neighbourhood search radius *d.* The neighbourhood definitions were the same as the global statistics were applied. Unlike the global Moran's *I,* which has the same expected value for the entire study area, the expected value of local Moran's *I* varies for each sampling location because it is calculated in relation to its particular set of neighbours.

$$E(I_i) = -\frac{1}{n-1}\sum_{j=1}^{n} W_{i,j}$$

The significance of the local Moran's *I* was calculated using a randomization test on the Z–score with 9,999 permutations to achieve highly significant values. Positive spatial autocorrelation occurs when, for example, a household with a specific outcome value is surrounded by neighbouring households with similar outcome value (low-low, high-high), thus forming a spatial cluster.

Results of the global analysis are shown in Table A1.

**References**


1. Moran, PAP. Notes on continuous stochastic phenomena. Biometrika. 1950;37:17-23.
2. Anselin L. Local Indicators of Spatial Association—LISA. Geographical Analysis. 1995;27:93-115.
3. Nelson TA, Boots B. Detecting spatial hot spots in landscape ecology. Ecography. 2008;31:556-66.
4. Cressie NAC. Statistics for Spatial Data. New York: John Wiley & Sons, 1993.
5. Fortin MJ, Dale MRT. Spatial analysis: a guide for ecologists. Cambridge: Cambridge University Press, 2014.

**Table A1.** Global univariate Moran's *I* values for socioeconomic variables in 100 households in Nagongera, Uganda

| Outcome | Moran's *I* | z-score | p-value |
|---|---|---|---|
| Wealth index score | 0.07 | 1.42 | 0.16 |
| Land area cultivated | -0.04 | -0.55 | 0.58 |
| House type[a] | -0.01 | -0.05 | 0.96 |

[a]House type: modern (cement, wood or metal walls; and tiled or metal roof; and closed eaves) or traditional (all other homes). All other variables were modelled as continuous.

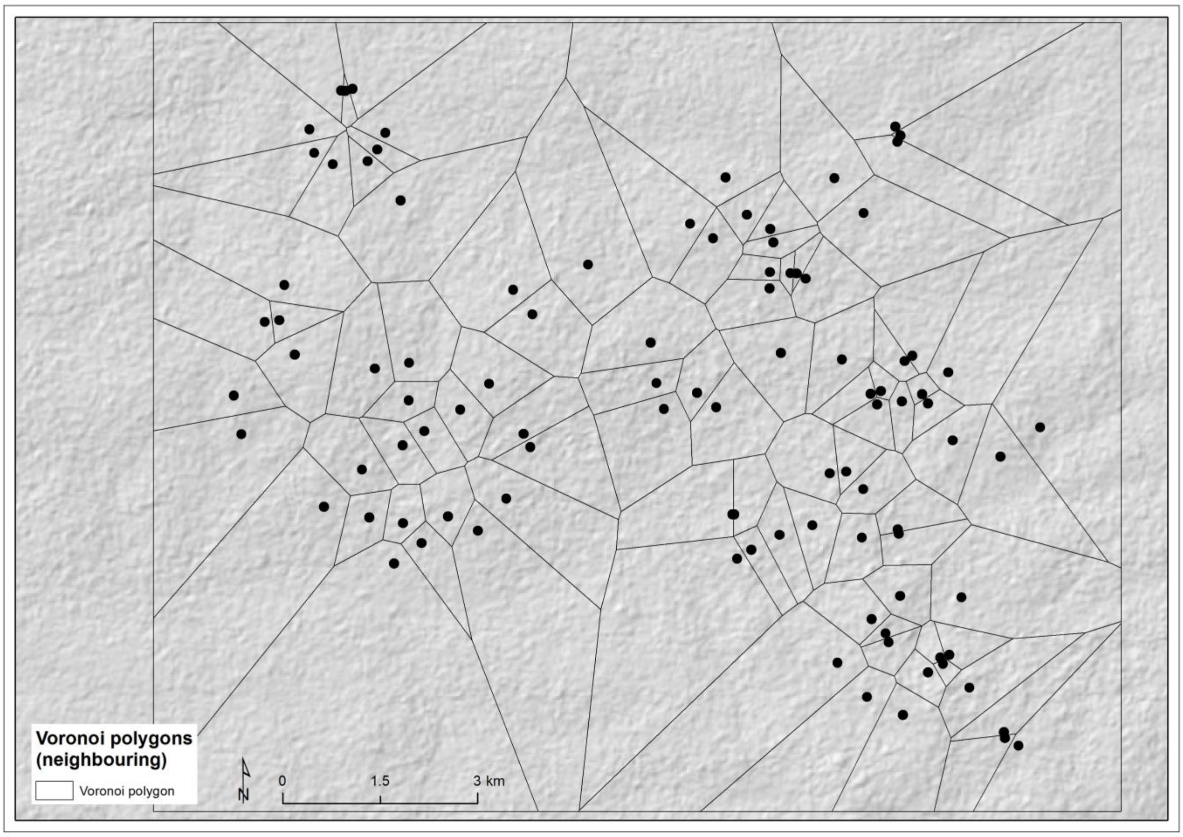


**Figure A1**. Neighbourhood matrix performed based on Delaunay triangulation to model the spatial relationship between households within the study area.
