# Supplementary material for: Why is malaria associated with poverty? Findings from a cohort study in rural Uganda
Source: Infect Dis Poverty. 2016 Aug 4;5:78. doi: 10.1186/s40249-016-0164-3 (PMC4972958; doi:10.1186/s40249-016-0164-3)
Supplement: Additional file 3: — Implementation of the mediation analysis. (PDF 280 kb) [file 40249_2016_164_MOESM3_ESM.pdf]

Additional File 2. Implementation of the mediation analysis

A mediator, *M,* is any factor that is on the causal pathway between an exposure, *X,* and an outcome, *Y*. Mediation analysis seeks to quantify the part of the total effect of *X* on *Y* that is explained by the effect through *M* (the indirect effect) and the part that does not occur through *M* (the direct effect). Here it is hypothesised that the relationship between socioeconomic position (SEP) (*X)* and malaria infection risk in children (*Y)* is mediated partly by three variables (*M*): caregiver's treatment-seeking behaviour, housing quality and food security. Assuming that malaria does not affect SEP (i.e. no reverse causality), we aimed to quantify the proportion of the total effect of SEP on malaria infection that was mediated by these three variables.

A simple approach to mediation analysis is to fit two regression models to estimate: (i) the effect of *X* on *Y*, adjusting for measured confounders, and (ii) the effect of *X* on *Y*, adjusting for measured confounders and *M*. An observed reduction in the magnitude of the effect estimate in the second model may be interpreted as evidence of mediation by *M*; in other words, that *M* explains part of the association between *X* and *Y.* This approach has been used to study how SEP affects tuberculosis risk, for example [1]. But it does not provide an estimate of the indirect effect.

One way to estimate the indirect effect is to assume that all the effects in a directed acyclic graph can be estimated by linear regression [2, 3]. The indirect effect is then the product of the effect of the exposure on the mediator and of the effect of the mediator on the outcome. The total effect is the direct effect plus the indirect effect. More recent methods allow for non-linear models [4-6]. Here we applied the Monte Carlo simulation approach described by Imai [7], which is flexible in being able to accommodate linear and nonlinear relationships, continuous and discrete mediators and various types of outcome variables. The following algorithm was used to estimate average causal mediation effects [7]:

1. Fit parametric models for the observed mediating and outcome variables.
2. Simulate model parameters from their sampling distributions.
3. Repeat the following three steps:
    a. *Simulate the potential values of the mediator:* Two potential values of the mediator are generated, each based on the mediator model, one under exposure and one under non-exposure.
    b. *Simulate the potential outcomes given the simulated values of the mediator*: For each exposure status two potential values of the outcome are generated, each based on the outcome model, one using the mediator value under exposure and one using the mediator value under non-exposure.
    c. *Compute the causal mediation effects:* The difference is taken between the two outcome predictions under exposure and the two outcome predictions under non-exposure. These differences are then averaged across all study units.
4. Compute summary statistics: The point estimate of the average causal mediation effect and its uncertainty estimates are computed from the distribution of mediation effects.

The average causal mediation effect estimates are only valid if two sequential ignorability assumptions are met [8, 7]: (i) conditional on the observed pretreatment covariates, the treatment is

independent of all potential values of the outcome and mediating variables and (ii) the observed mediator is independent of all potential outcomes given the observed treatment and pretreatment variables. In practice, these will hold if there is no unmeasured confounding of the association between exposure and mediator, exposure and outcome or mediator and outcome, and there is no reverse causality.

We implemented the algorithm using the *medeff* command with 1000 simulations in Stata13 (StataCorp, Texas) [9], to calculate the effect of SEP on malaria infection risk mediated by house type and food security. Treatment-seeking behaviour was not assessed as a potential mediator due to missing data. Age and gender were included as covariates and we adjusted for clustering at the level of the household.

Limitations
While the identification of potential mediators between SEP and malaria provides evidence of a biologically plausible mechanism for causality, the mediation analysis had a number of limitations. First, it is unlikely that the assumption of no reverse causality was met. Reverse causality from malaria to poverty in Nagongera is highly probable, since the direct and indirect costs of malaria may cause poverty within households, as observed in Tanzania [10, 11].

Second, it is not possible to exclude the possibility of unmeasured confounding between exposure and mediator and between mediator and outcome. In particular, the assumption that the mediator is ignorable given observed treatment and pretreatment confounders (i.e. that among children in the same category of SEP and with the same pretreatment characteristics, the mediator can be regarded as if it were randomised) is very strong. Even in randomised studies it is always possible that there is unmeasured confounding between mediator and outcome. In our study there may have been confounding of the association between house type and malaria, for example, by distance of house to village periphery among numerous other factors [12]. Furthermore, using the Imai algorithm it is not possible to control for confounders of the mediator-outcome relationship, even if these are measured, without an additional assumption [7]. Therefore, regardless of the number of confounders measured, it is difficult to establish the ignorability of the mediator.

Third the mediation analysis investigated only three potential mediators of the SEP-malaria relationship and accounted for less than half of the total effect. This suggests that other mediating factors remained unaccounted for. Finally, it is not clear that the study was adequately powered for the mediation analysis, the sample size (N = 300) being calculated to compare temporal changes in malaria incidence from the cohort with temporal changes in malaria test positivity rate from health facility based surveillance [13].

References

1. Boccia D, Hargreaves J, De Stavola B, Fielding K, Schaap A, Godfrey-Faussett P et al. The association between household socioeconomic position and prevalent tuberculosis in Zambia: a case-control study. PLoS ONE. 2011;6:e20824.
2. Wright S. The method of path coefficients. Annals Mathematical Statistics. 1934;5:161-215.
3. Bollen KA, Stine RA. Direct and indirect effects: Classical and bootstrap estimates of variability. Sociological Methodology. 1990;20:115-40.
4. VanderWeele TJ, Vansteelandt S. Odds ratios for mediation analysis for a dichotomous outcome. Am J Epidemiol. 2010;172:1339-48.
5. VanderWeele TJ. Causal mediation analysis with survival data. Epidemiology. 2011;22:582-5.
6. Vansteelandt S, Bekaert M, Lange T. Imputation strategies for the estimation of natural direct and indirect effects. Epidemiologic Meth. 2012;1:131-58.
7. Imai K, Tingley D, Keele L. A general approach to causal mediation analysis. Psychological Meth. 2010;15:309-34.
8. Imai K, Keele L, Yamamoto T. Identification, inference, and sensitivity analysis for causal mediation effects. Statistical Science. 2010;25:51-71.
9. Hicks R, Tingley D. Mediation: stata package for causal mediation analysis. 2011.
10. Somi MF, Butler JRG. Is there evidence for dual causation between malaria and socioeconomic status? Findings from rural Tanzania. Am J Trop Med Hyg. 2007;77:1020-7.
11. de Castro MC, Fisher MG. Is malaria illness among young children a cause or a consequence of low socioeconomic status? Evidence from the United Republic of Tanzania. Malar J. 2012;11:161.
12. Russell T, Lwetoijera D, Knols B, Takken W, Killeen G, Kelly-Hope L. Geographic coincidence of increased malaria transmission hazard and vulnerability occurring at the periphery of two Tanzanian villages. Malar J. 2013;12(1):24.
13. Kamya MR, Arinaitwe E, Wanzira H, Katureebe A, Barusya C, Kigozi SP et al. Malaria transmission, infection and disease at three sites with varied transmission intensity in Uganda: implications for malaria control. Am J Trop Med Hyg. 2015;92:903-12.
